# Stochastic Optimization for an Analytical Model of Saltwater Intrusion in Coastal Aquifers

**DOI:** 10.1371/journal.pone.0162783

**Published:** 2016-09-30

**Authors:** Paris N. Stratis, George P. Karatzas, Elena P. Papadopoulou, Maria S. Zakynthinaki, Yiannis G. Saridakis

**Affiliations:** 1 School of Production Engineering and Management, Technical University of Crete, Chania 73100, Greece; 2 School of Environmental Engineering, Technical University of Crete, Chania 73100, Greece; 3 School of Mineral Resources, Technical University of Crete, Chania 73100, Greece; 4 Applied Mathematics & Computers Laboratory, Technical University of Crete, Chania 73100, Greece; Centro de Investigacion Cientifica y de Educacion Superior de Ensenada Division de Fisica Aplicada, MEXICO

## Abstract

The present study implements a stochastic optimization technique to optimally manage freshwater pumping from coastal aquifers. Our simulations utilize the well-known sharp interface model for saltwater intrusion in coastal aquifers together with its known analytical solution. The objective is to maximize the total volume of freshwater pumped by the wells from the aquifer while, at the same time, protecting the aquifer from saltwater intrusion. In the direction of dealing with this problem in real time, the ALOPEX stochastic optimization method is used, to optimize the pumping rates of the wells, coupled with a penalty-based strategy that keeps the saltwater front at a safe distance from the wells. Several numerical optimization results, that simulate a known real aquifer case, are presented. The results explore the computational performance of the chosen stochastic optimization method as well as its abilities to manage freshwater pumping in real aquifer environments.

## Introduction

### The present study

Saltwater intrusion in freshwater aquifers is a major concern in coastal areas around the globe. Apart from natural disastrous phenomena, such as earthquakes or floods, human activities and pumping policies nearby the coastline can seriously affect the chemical constitution of water aquifers.

Under predevelopment conditions, groundwater systems are in long-term equilibrium (cf. [1–3]). That is, averaged over some period of time, the amount of water entering or recharging the system is approximately equal to the amount of water leaving or discharging from the system. Water discharges and recharges are more or less equal and the total volume of the water within the aquifer remains almost constant. Groundwater levels fluctuate in time over a relatively modest, natural range. Once pumping begins, this equilibrium changes. The main factors for this are the consumptive water use and the irrigation for agricultural needs at nearby areas, that peak during summer months.

People’s access to high quality water is a priority for the UN not only due to the climate changes effects, especially severe in coastal regions, but also due to the “unfair” allocation of water resources. The increased worldwide concern to protect groundwater resources in coastal regions is a motivation for the research community to better understand the behavior of complex physical systems and explore new areas of optimization that will improve the evaluation of integrated management solutions as they are absolutely essential to maintain freshwater quality and avoid all the economical implications from salinization of freshwater aquifers. Scientific and engineering community’s interest in this problem remains unrelenting for over a century (cf. [4–8] for a review) and has increased over the recent years, as the demand for optimal solutions becomes eminent.

Water flow inside an aquifer is a complex process, as two mixing fluid phases exist and the hydraulic parameters of the aquifer share a great spatial variability (cf. [6]). Several factors, such as the difficulty and uncertainty in generating reliable estimates of the problem’s parameters as well as the computational cost, have led to model simplifications. The *sharp-interface* simplification, based basically on the *Ghyben-Herzberg* relation, require fewer parameters and, in certain situations (cf. [9]), provide reasonably good approximations. Thus, it has been studied and used extensively in the literature (e.g. cf. [10–19]) coupled with optimization algorithms in forming simulation-optimization models for groundwater management problems. The optimization algorithms used in these models are based on many different techniques such as sequential quadratic programming (cf. [14, 15]), genetic algorithms (cf. [20–24]), evolutionary algorithms (cf. [15, 25]), simulated annealing (cf. [26]) and differential evolution (cf. [27]).

In the present study the sharp-interface model [2] for saltwater intrusion in coastal unconfined aquifers of finite size and its analytical solution (cf. [14]) are used as foundation tools for the optimal management of freshwater pumping by means of stochastic optimization. Our main objective is to produce a simple method able to rapidly *maximize the total pumping rate* of the aquifer wells, and, at the same time, provide full control over both the *safety of the aquifer wells* and the *pumping policies*, as far as salinization is concerned. For this, we adapt ALOPEX (cf. [28]) stochastic unconstrained optimization procedure coupled by a penalty system, that implements the necessary constraint conditions to avoid saltwater intrusion, to guide and manage the pumping process. The behavior and the computational efficiency of the whole optimization procedure are being investigated through the examination of several pumping and weather-soil conditions on a rectangular shaped aquifer. Our test cases, presented here, approximate a known problem from the literature (cf. [14, 15]) which simulates a real aquifer at Vathy area of the Greek island of Kalymnos.

### ALOPEX stochastic optimization

ALOPEX (ALgorithm Of Pattern EXtraction [28, 29]) is a stochastic iterative process seeking the optimum values of the control variables, in order to maximize an objective (profit, cost, energy) function. It is based on a balanced combination of a bias feedback term and a stochastic term to update the values of the control variables. The bias term uses local correlations between changes in individual control variables as well as changes in the global error measure and has the tendency to move each control variable to the direction which was successful in the past. The stochastic term is a random number, generated for each control variable in each iteration, and provides the opportunity to move each variable against the direction of recent success and avoid local extrema. Balance between the bias and the stochastic term is absolutely necessary as the method reduces to a simple gradient descent in case the stochastic term is omitted, and to a simple random walk in case the bias term is omitted.

ALOPEX was originally devised and introduced in [28] (see also [29]) for the purpose of experimentally determining receptive fields of individual neurons in the visual pathway. Later on, the method was studied in detail and new versions were introduced aiming in accelerating its convergence (cf. [30] and [31] for example). Application areas, where the method was successfully applied, include neurophysiology [28], pattern recognition (cf. [32]), image enhancement in adaptive optics (cf. [33]), cardiovascular or biomechanical applications (cf. [34]) and neural networks (cf. [32, 35–37]). Some preliminary and limited results pertaining to the utilization of the ALOPEX II form for coastal aquifers may be found in [38]. The method, through its applications has been compared with known methods, demonstrating success in variety of problems and showing specific advantages over other optimization techniques (cf. [39]).

ALOPEX is an easily implemented stochastic algorithm and presents an alternative to Simulated Annealing [40] (SA). Some of its main features include:

- Requires no knowledge of the dynamics of the system, it is essentially gradient-free, and it does not explicitly depend on the functional form of the objective function but rather on estimates of its values in previous steps. This fact alone has a major contribution to the applicability of the algorithm in real time environments and its implementation by use of analog or digital devices (cf. [41]).
- Updates simultaneously all control variables, while SA does not. Each variable is computed using only local computations and independently of the other variables. The computation of the whole process can be carried out completely in parallel (cf. [42]) contributing to its computational efficiency.
- The magnitude of the stochastic term does not depend on the amount it raises or lowers the response, as in SA via the Boltzmann distribution. This makes it easier for the variables to traverse wide gaps between extrema. However, if its magnitude is much higher than the magnitude of the bias term, then the process is mainly driven by randomness, a situation similar to the *melting* process in SA.

Coastal aquifer management problems are formed as optimization problems with typically nonlinear objectives and constraints. Stochastic, heuristic and adaptive search algorithms, like ALOPEX, SA or Genetic Algorithms, have been developed and suggested for problems with nonlinear objectives and constraints, mainly to overcome the difficulties gradient-based methods are facing with local extrema. Adding to this the ease of implementation and low computational cost of the ALOPEX stochastic algorithm, we believe that it might be potentially useful to the engineering community.

## Materials and Methods

**Fig 1:**
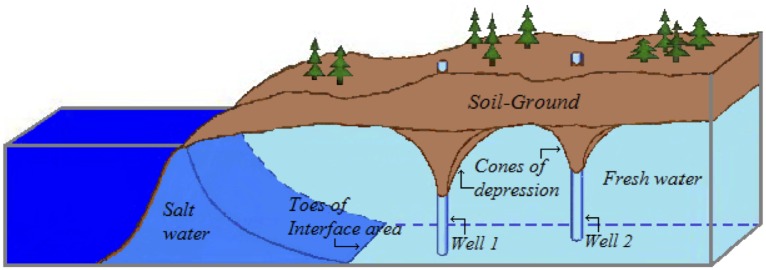
Schematic representation of salt water intrusion under sharp-interface and Ghyben-Herzberg assumptions.

As we have already mentioned, the model we consider for the saltwater intrusion problem in coastal unconfined aquifers of finite size, is based on the *sharp-interface* simplification, which assumes that there is no mixing (transition) zone between freshwater and saltwater inside the aquifer (see Fig 1), and on the *Ghyben-Herzberg* relation, which at steady state estimates the position of the interface assuming that horizontally floating freshwater floats over static saltwater. The sharp-interface approximation is reasonable in regional scale problems when the transition zone is narrow relative to the scale of the problem (cf. [9, 15]). In this situation, the approximation is more appropriate depending on the hydro-geological parameters, the aquifer geometry (especially the layer thickness), the location of the pumping wells and the recharge/pumping scenarios, as it has been recently studied in [9]. For the model aquifer we consider in this work it will be shown, in later sections (in particular in section that investigates validity issues of the optimization results), that there are ranges of the hydro-geological parameters that narrow the width of the transition zone without compromising the stability of the whole computation. In this sense, one may regard the model aquifer as an appropriate one to be studied under the sharp interface and the Ghyben-Herzberg assumptions. In the section that follows, the governing equation of the sharp-interface approach and its analytical solution are described.

### Model equations based on a single potential formulation

**Fig 2:**
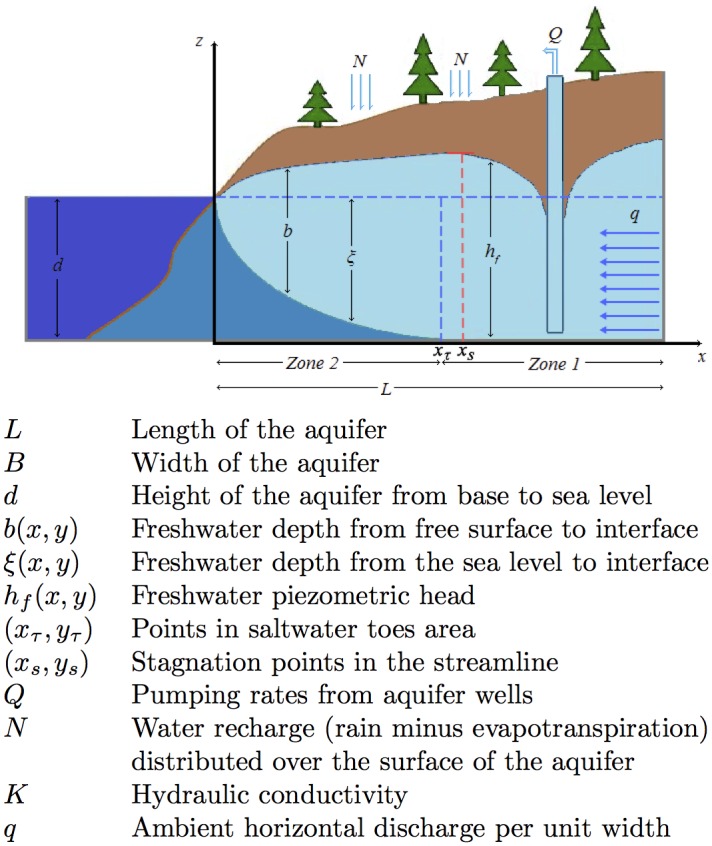
Cross section of a coastal freshwater aquifer. Modelling parameters.

Consider the unconfined aquifer, depicted in Figs 1 and 2, and observe that the interface between saltwater and freshwater areas is assumed sharp. Freshwater movement from inland towards the coastline repels saltwater towards the sea. When the total discharge rate *Q* increases due to pumping activities, saltwater toe penetration *x*_*τ*_ moves towards the interior of the aquifer, while, at the same time, the *stagnation* point *x*_*s*_, that separates water flowing into the sea from water flowing into the well, moves towards the coastline (see Fig 2). And whenever *Q* overpasses a critical value *Q*_*c*_, toe penetration *x*_*τ*_ crosses over the stagnation point *x*_*s*_ salinizing the well. Clearly, therefore, the detection of the saltwater wedge toes *x*_*τ*_ would provide a measure for the distance of saltwater interface from the active pumping sources (wells) and alarm us for the danger of pumping salinized water.

Working towards this direction, Strack (1976, cf. [43]), considering the sharp-interface approximation and the Ghyben-Herzberg relation, as well as the Dupuit hydraulic assumption, derived a two dimensional single-potential model and used it, together with the method of images, to analytically describe the saltwater interface. Since then, many authors have been also used the single potential formulation (cf. [8, 14–16, 19, 21, 44, 45]) to study saltwater intrusion.

To briefly describe Strack’s model and fix notation, let us first consider the Ghyben-Herzberg relation:

$$h_{f}-d=\delta \;\xi \;\;,\;\;\;\;\delta :=\frac{\rho_{s}-\rho_{f}}{\rho_{f}}\approx 0.025$$ (1)

where *ρ*_*s*_ and and *ρ*_*f*_ denote the density of salt and fresh water, respectively. Moreover, it is well known that the governing equation of steady flow in an unconfined aquifer can be expressed as (cf. [2, 14]):

$$\frac{\partial }{\partial x}\left(Kb\frac{\partial h_{f}}{\partial x}\right)+\frac{\partial }{\partial y}\left(Kb\frac{\partial h_{f}}{\partial y}\right)+N-Q=0,$$ (2)

where *b* satisfies

$$\begin{array}{c} \text{Zone}\;\text{1:}\;b=h_{f},\\ \text{Zone}\;\text{2:}\;b=h_{f}-d+\xi . \end{array}$$ (3)

Then, Strack’s flow potential *ϕ* = *ϕ*(*x*, *y*), defined by (cf. [43]):

$$\begin{array}{c} \text{Zone}\;\text{1:}\;\phi =\frac{1}{2}\left(h_{f}^{2}-\left(1+\delta \right)d^{2}\right),\\ \text{Zone}\;\text{2:}\;\phi =\frac{1+\delta }{2\delta }{\left(h_{f}-d\right)}^{2}, \end{array}$$ (4)

is a continuous and smooth function across the boundary between zones 1 and 2, satisfying the differential equation

$$\frac{\partial }{\partial x}\left(K\frac{\partial \phi }{\partial x}\right)+\frac{\partial }{\partial y}\left(K\frac{\partial \phi }{\partial y}\right)+N-Q=0\;\;,$$ (5)

with Dirichlet boundary conditions *ϕ* = 0 at the coast boundary (*x* = 0) and Neumann boundary conditions *ϕ*_*η*_ = 0 at the no flow boundaries (*η* is the normal vector). Furthermore, at the location of the interface toes, where *ξ* = *d* hence *h*_*f*_ = (1 + *δ*)*d* from relation Eq (1), there holds

$$\phi_{\tau }=\phi \left(x_{\tau },y_{\tau }\right)=\frac{(1+\delta )\delta }{2}d^{2}\;.$$ (6)

If, now, the values of *K*, *N*, *Q* and the boundary conditions are known, the differential Eq (5) can be solved for *ϕ*(*x*, *y*) using analytical or numerical methods. Once *ϕ* = *ϕ*(*x*, *y*) is determined, the position *z* = *z*(*x*, *y*) of the interface surface, as well as the piezometric surface *h*_*f*_, can be calculated as a function of *ϕ*, by

$$\begin{array}{c} \text{Zone}\;\text{1:}\;z=0,\;h_{f}=\sqrt{2\phi +\left(1+\delta \right)d^{2}},\;\text{for}\;\frac{(1+\delta )\delta }{2}d^{2}\leq \phi ,\\ \text{Zone}\;\text{2:}\;z=d-\xi ,\;\xi =\sqrt{\frac{2\phi }{\delta (1+\delta )}}\;\text{and}\;h_{f}=\sqrt{\frac{2\delta \phi }{1+\delta }}+d,\;\text{for}\;0\leq \phi \leq \frac{(1+\delta )\delta }{2}d^{2}, \end{array}$$ (7)

and the locus of the toes of the interface area can be determined by solving, for *x*_*τ*_, the nonlinear equation described in relation Eq (6) above.

### Analytical solution

**Fig 3:**
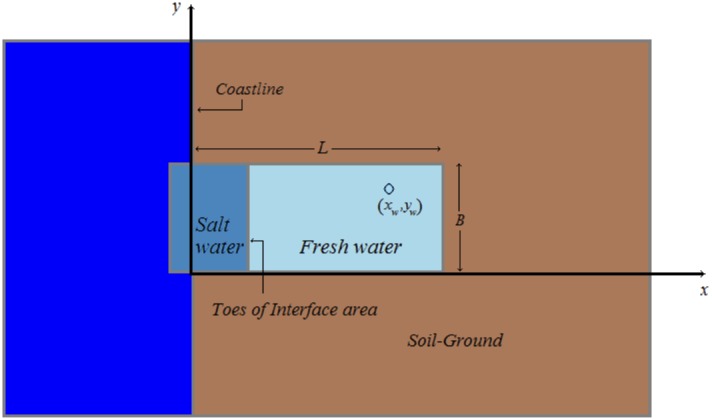
Homogeneous rectangular aquifer.

Let us now consider the case of homogeneous (constant hydraulic conductivity *K*) rectangular aquifers of finite size with a fixed head boundary at the coastline, and three other impermeable boundaries, as it is visually demonstrated in Fig 3 that follows.

For this case, Mantoglou [14], using the method of images and superposition to incorporate both ambient flow and surface accretion, extended Strack’s work and presented a generalized analytical solution of the differential Eq (5). The analytical representation of the flow potential *ϕ* = *ϕ*(*x*, *y*) included in [14] and used in our simulations is given by:

$$\begin{array}{ccc} \phi (x,y) & = & \frac{q}{K}x+\frac{N}{K}x\left(L-\frac{x}{2}\right)+\sum_{k=1}^{2}\;\sum_{i=1}^{3}\;\sum_{j=1}^{M}\frac{Q_{j}}{4\pi K}ln\left(\frac{a_{2i-1,j}\left(x\right)+b_{k,j}\left(y\right)}{a_{2i,j}\left(x\right)+b_{k,j}\left(y\right)}\right)+\\ & & +\sum_{n=1}^{2}\;\sum_{k=3}^{6}\;\sum_{i=1}^{3}\;\sum_{j=1}^{M}\frac{Q_{j}}{4\pi K}ln\left(\frac{a_{2i-1,j}\left(x\right)+b_{k,n,j}\left(y\right)}{a_{2i,j}\left(x\right)+b_{k,n,j}\left(y\right)}\right) \end{array}$$ (8)

where

$$\begin{array}{ccc} a_{1,j}\left(x\right):={\left(x-x_{j}\right)}^{2} & \; & b_{1,j}\left(y\right):={\left(y-y_{j}\right)}^{2}\\ a_{2,j}\left(x\right):={\left(x+x_{j}\right)}^{2} & \; & b_{2,j}\left(y\right):={\left(y+y_{j}\right)}^{2}\\ a_{3,j}\left(x\right):={\left(x-\left(2L-x_{j}\right)\right)}^{2} & \; & b_{3,n,j}\left(y\right):={\left(y-\left(2nB-y_{j}\right)\right)}^{2}\\ a_{4,j}\left(x\right):={\left(x-\left(2L+x_{j}\right)\right)}^{2} & \; & b_{4,n,j}\left(y\right):={\left(y-\left(2nB+y_{j}\right)\right)}^{2}\\ a_{5,j}\left(x\right):={\left(x+\left(2L+x_{j}\right)\right)}^{2} & \; & b_{5,n,j}\left(y\right):={\left(y+\left(2nB-y_{j}\right)\right)}^{2}\\ a_{6,j}\left(x\right):={\left(x+\left(2L-x_{j}\right)\right)}^{2} & \; & b_{6,n,j}\left(y\right):={\left(y+\left(2nB+y_{j}\right)\right)}^{2} \end{array}$$ (9)

and *Q*_*j*_ denotes the pumping rate (m^3^/day) of the *j*-th aquifer well *w*_*j*_, *j* = 1, …, *M*, with coordinates (*x*_*j*_, *y*_*j*_) and considered to be non-negative (*Q*_*j*_ ≥ 0). We point out that, throughout this paper, the wells are assumed to be numbered in a way such that *x*_1_ ≤ *x*_2_ ≤ ⋯ ≤ *x*_*M*_.

## Pumping Management

As we have already mentioned in previous sections, our main objective is to maximize the total volume of fresh water pumped by the wells while keeping at the same time the wells safe from saltwater intrusion. To numerically simulate said task, let us define the normalized objective (or profit) function *P*: ℝ^*M*^ → ℝ as

$$P\equiv P\left(Q_{1},\ldots ,Q_{M}\right)=e^{-{\left(1-\frac{\sum_{j=1}^{M}Q_{j}}{\sum_{j=1}^{M}{\bar{Q}}_{j}}\right)}^{2}}\in \left(0,1\right]$$ (10)

with

$$0\leq {\underline{Q}}_{j}\leq Q_{j}\leq {\bar{Q}}_{j}<{\bar{Q}}_{A}\;\;,\;\;\;j=1,\ldots ,M$$ (11)

and

$$\sum_{j=1}^{M}Q_{j}\leq {\bar{Q}}_{A}$$ (12)

where ${\underline{Q}}_{j}$ and ${\bar{Q}}_{j}$ denote the minimum and maximum, respectively, pumping rate (m^3^/day) capabilities of the *j*th-well and may depend on several technical parameters (e.g. pumping equipment, pipes, hoses) as well as on local pumping policies imposed to regulate social, agricultural or industrial needs, while ${\bar{Q}}_{A}$ denotes the maximum volume (m^3^/day) of fresh water available for pumping from the aquifer.

Observe that, if we let

$$Q={\left[Q_{1}\;\cdots \;Q_{M}\right]}^{\text{T}}\;\text{and}\;S\equiv S\left(Q\right)=\sum_{j=1}^{M}Q_{j}\;,$$ (13)

then we may also write the objective function as

$$P\equiv P\left(Q\right)\equiv P\left(S\left(Q\right)\right)=e^{-{\left[S\left(\bar{Q}\right)-S\left(Q\right)\right]}^{2}/S^{2}\left(\bar{Q}\right)}$$ (14)

where, of course, $\bar{Q}={\left[{\bar{Q}}_{1}\;\ldots \;{\bar{Q}}_{M}\right]}^{\text{T}}$ denotes the vector of maximum pumping rates.

It can be, now, easily verified that, as

$$\frac{\partial P(Q)}{\partial Q_{i}}=\frac{2}{S^{2}\left(\bar{Q}\right)}\left[S\left(\bar{Q}\right)-S\left(Q\right)\right]P\left(Q\right)\;\;,\;\;\;i=1,\ldots ,M\;\;,$$ (15)

hence

$$\frac{\partial P(Q)}{\partial Q_{i}}=0\;\text{for}\;S\left(Q\right)=S\left(\bar{Q}\right)\;\;\text{and}\;\;\text{sign}\left(\frac{\partial P(Q)}{\partial Q_{i}}\right)=\text{sign}\left(S\left(\bar{Q}\right)-S\left(Q\right)\right)\;,$$ (16)

the basic properties of the objective function are described by:

$$P\left(Q\right)↑\;\text{for}\;S\left(Q\right)<S\left(\bar{Q}\right)$$ (17)

$$P\left(Q\right)↓\;\text{for}\;S\left(Q\right)>S\left(\bar{Q}\right)$$ (18)

$$\nabla P\left(Q\right)=0\;\text{for}\;S\left(Q\right)=S\left(\bar{Q}\right)\;.$$ (19)

Therefore, the objective function *P*(***Q***) attains its maximum

$$\max_{Q}P\left(Q\right)=1\;\text{whenever}\;S\left(Q\right)=S\left(\bar{Q}\right)\;.$$ (20)

**Fig 4:**
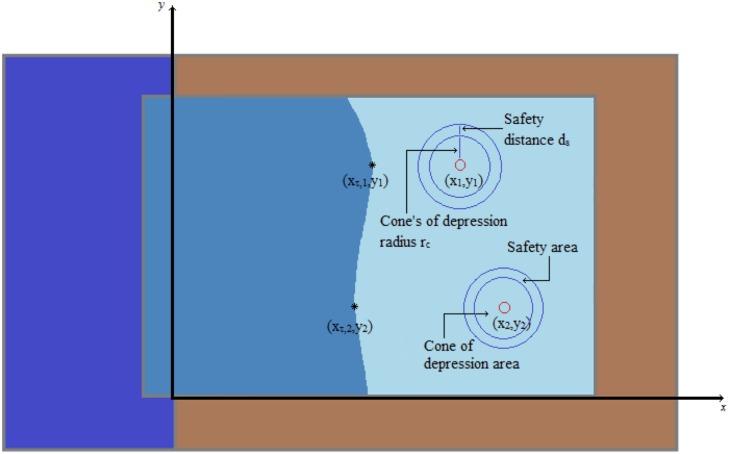
Cone of depression and safety distance areas around every aquifer well.

To protect, now, the wells from saltwater intrusion, we couple the objective function *P*(***Q***) with a reinforced *Toe* constraint. For this, consider two circular areas around every well, shown schematically in Fig 4 (cf. [38]). The inner circular area represents the area of the *cone of depression*, where water-pumping results in an accelerated movement of the water in this area towards the well. The radius of the cone’s of depression area is denoted by *r*_*c*_ and, in the literature, varies from 200m to 1200m depending on the maximum well pumping rates and the aquifer natural recharge capabilities. The outer circular area represents a safety distance barrier around the cone of depression of radius *r*_*c*_ + *d*_*s*_, with *d*_*s*_ > 0, to prevent saltwater reaching the cone’s of depression area. Then, the Toe constraint, which couples the objective function *P*, takes the form

$$x_{\tau ,j}\equiv x_{\tau ,j}\left(Q_{1},\ldots ,Q_{M}\right)\leq x_{j}-\left(r_{c}+d_{s}\right)\;\;,\;j=1,\ldots ,M\;,$$ (21)

where (*x*_*τ*,*j*_, *y*_*j*_) denotes the saltwater front’s point across the *j*-th aquifer well *w*_*j*_ with coordinates (*x*_*j*_, *y*_*j*_). We remark that it might be necessary, especially for aquifers with more than one barriers at the sea, to consider a small number of adjacent points (e.g. on the circle of radius *r*_*c*_ + *d*_*s*_) to effectively protect the wells from saltwater intrusion. Finally, we would like to point out that the toe constraint condition may also be coupled (cf. [15]) by the potential constraints

$$\phi (x_{j},y_{j})>0\;\;,\;j=1,\ldots ,M\;,$$ (22)

which force the free surface of the aquifer to be maintained above the sea level. These constraints mainly protect wells located sufficiently far from the coast (cf. [15]). In this work we will adopt them, together with the rest of the constraints, in an attempt to resolve certain stability issues of the optimization results.

## ALOPEX for optimized pumping management

Following last section’s discussion, pumping management can be expressed in the following nonlinear optimization form:

$$\begin{array}{ll} \text{Determine} & \\ & \;\;\underset{Q}{\text{max}}P\left(Q\right)\\ \text{under the constraints} & \\ & \begin{array}{l} {\underline{Q}}_{j}\leq Q_{j}\leq {\bar{Q}}_{j}\;,\;j=1,\ldots ,M\\ S\left(Q\right)\leq {\bar{Q}}_{A}\\ x_{\tau ,j}\leq x_{j}-\left(r_{c}+d_{s}\right)\;,\;j=1,\ldots ,M\;. \end{array} \end{array}$$ (23)

We will also consider the case of reinforcing the above set of constraints by the potential constraint, described in Eq (22), namely

$$\phi (x_{j},y_{j})>0\;\;,\;j=1,\ldots ,M\;,$$

and will report separately on its effect on the total pumping rate.

### ALOPEX optimization procedure

For the solution of the above problem we consider the employment of the ALOPEX stochastic optimization method along with appropriate penalty conditions to implement the constraints described above. Taking into account the fact that the objective function is already normalized, among the several different variations of ALOPEX stochastic optimization a modified ALOPEX II version (cf. [31]) is adopted and used in our simulations. In each iteration step of this ALOPEX stochastic process all control variables *Q*_*j*_, *j* = 1, …, *M*, are updated simultaneously by means of the following vector rule

$$Q^{\left(k\right)}=Q^{\left(k-1\right)}+c_{k}\Delta P^{\left(k-1\right)}{\Delta Q}^{\left(k-1\right)}+g^{\left(k\right)}\;,\;\;k=2,3,\ldots$$ (24)

with

$$\begin{array}{c} {\Delta Q}^{\left(k\right)}=Q^{\left(k\right)}-Q^{\left(k-1\right)}\\ \Delta P^{\left(k\right)}=P\left(Q^{\left(k\right)}\right)-P\left(Q^{\left(k-1\right)}\right) \end{array}$$ (25)

provided, of course, initial values of the control variables ***Q***^(*k*)^ for *k* = 0, 1. Furthermore, *c*_*k*_ is a real parameter controlling the amplitude of the feedback term, while ***g***^(*k*)^ is the noise vector, with values uniformly distributed in an appropriately chosen interval, to provide the necessary agitation needed to drive the process to global extrema avoiding local problems. As we further discuss in Remarks 2 and 3 at the end of this section, the values *c*_*k*_ and $g_{j}^{\left(k\right)}$, attained throughout the whole ALOPEX process, are given by

$$c_{k}=\frac{1}{|\Delta P^{\left(k-1\right)}|}$$ (26)

and

$$g_{j}^{\left(k\right)}=\gamma Q_{j}^{\left(k-1\right)}X_{j}^{\left(k\right)}\;,\;\;j=1,\cdots ,M$$ (27)

where *γ* denotes a small percentage 1% − 2% of $Q_{j}^{\left(k-1\right)}$ (here we use *γ* = 0.015) and $X_{j}^{\left(k\right)}$ is a random variable with values uniformly distributed in the interval (−0.5, 1). We remark that, with *c*_*k*_ defined as in Eq (26) above, the feedback term of the ALOPEX rule in Eq (24) reduces to *c*_*k*_ Δ*P*^(*k*−1)^ **Δ*Q***^(*k*−1)^ = sign(Δ*P*^(*k*−1)^)**Δ*Q***^(*k*−1)^, while the noise term corresponds to a few m^3/^day.

The next paragraph is being devoted to the development of a simple and effective deterministic frame of rectifications applied to the main ALOPEX step, described in relations Eqs (24) and (25) above, in order to implement the constraints described in Eq (23).

### Constraints implementation

In each iteration step *k* = 2, 3, …, the values of the control variables $Q_{j}^{\left(k\right)}$, *j* = 1, …, *M*, obtained by the ALOPEX relationship in Eq (24), are examined to satisfy the constraints described in Eq (23). In case of violation, an appropriate *penalty* strategy is implemented by means of the following rectifications:

∘ *Rectification for local minimum pumping rate constraint violation*

Whenever the current pumping rate $Q_{p}^{\left(k\right)}$ of the *p*-well, *p* ∈ {1, 2, …, *M*}, obtained by relationship Eq (24), violates the minimum pumping rate constraint, namely

$$Q_{p}^{\left(k\right)}<{\underline{Q}}_{p}\;\;\text{for}\;\;p\in \left\{1,2,\ldots ,M\right\}\;\;,$$

then the value of $Q_{p}^{\left(k\right)}$ is being rectified by

$$Q_{p}^{\left(k\right)}=\lambda {\underline{Q}}_{p}\;\;\text{for}\;\;p\in \left\{1,2,\ldots ,M\right\}\;\;,$$ (28)

where λ ≥ 1 is a real parameter.

∘ *Rectification for local maximum pumping rate constraint violation*

Whenever the current pumping rate $Q_{p}^{\left(k\right)}$ of the *p*-well, *p* ∈ {1, 2, …, *M*}, obtained by relationship Eq (24), violates the maximum pumping rate constraint, namely

$$Q_{p}^{\left(k\right)}>{\bar{Q}}_{p}\;\;\text{for}\;\;p\in \left\{1,2,\ldots ,M\right\}\;\;,$$

then the value of $Q_{p}^{\left(k\right)}$ is being rectified by

$$Q_{p}^{\left(k\right)}=\mu {\bar{Q}}_{p}\;\;\text{for}\;\;p\in \left\{1,2,\ldots ,M\right\}\;\;,$$ (29)

where 0 ≤ *μ* ≤ 1 is a real parameter.

∘ *Rectification for global maximum pumping rate constraint violation*

To ensure no violation of the maximum volume of fresh water available from the aquifer constraint, described in Eq (12), will occur at any time during the optimization procedure, let us assume that *k* − 1 iteration steps (*k* ≥ 2) of the optimization process have been carried out successfully (that is without any constraint violation or appropriately rectified). Furthermore, during the current *k* iteration step, assuming that all values $Q_{i}^{\left(k\right)}$, *i* = 1, …, *p* − 1 and 1 ≤ *p* ≤ *M*, have been successfully evaluated, let $S_{p-1}^{\left(k\right)}$ be defined by

$$S_{p-1}^{\left(k\right)}\equiv S\left(Q_{1}^{\left(k\right)},\cdots ,Q_{p-1}^{\left(k\right)},Q_{p}^{\left(k-1\right)},\cdots ,Q_{M}^{\left(k-1\right)}\right)=\sum_{i=1}^{p-1}Q_{i}^{\left(k\right)}+\sum_{i=p}^{M}Q_{i}^{\left(k-1\right)}\;\;,$$ (30)

with $S_{0}^{\left(k\right)}\equiv S_{M}^{\left(k-1\right)}\equiv S_{M}^{\left(k-1\right)}=S\left(Q_{1}^{\left(k-1\right)},\ldots ,Q_{M}^{\left(k-1\right)}\right)$. Upon evaluation of $Q_{p}^{\left(k\right)}$ pumping rate of the *p*-well, by relationship Eq (24), the global maximum pumping constraint is being violated whenever

$$\tilde{S}:=S_{p-1}^{\left(k\right)}+\Delta Q_{p}^{\left(k\right)}-{\bar{Q}}_{A}>0$$ (31)

in which case the value of $Q_{p}^{\left(k\right)}$ is being rectified by

$$Q_{p}^{\left(k\right)}=Q_{p}^{\left(k\right)}-\nu \tilde{S}\;\;,$$ (32)

where *ν* ≥ 1 is a real parameter.

**Fig 5:**
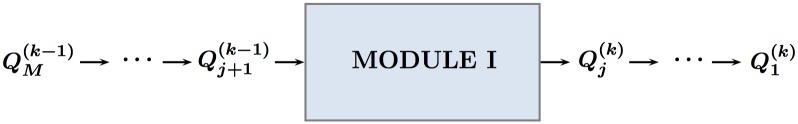
Pipelined Data Flow through MODULE I.

**Fig 6:**
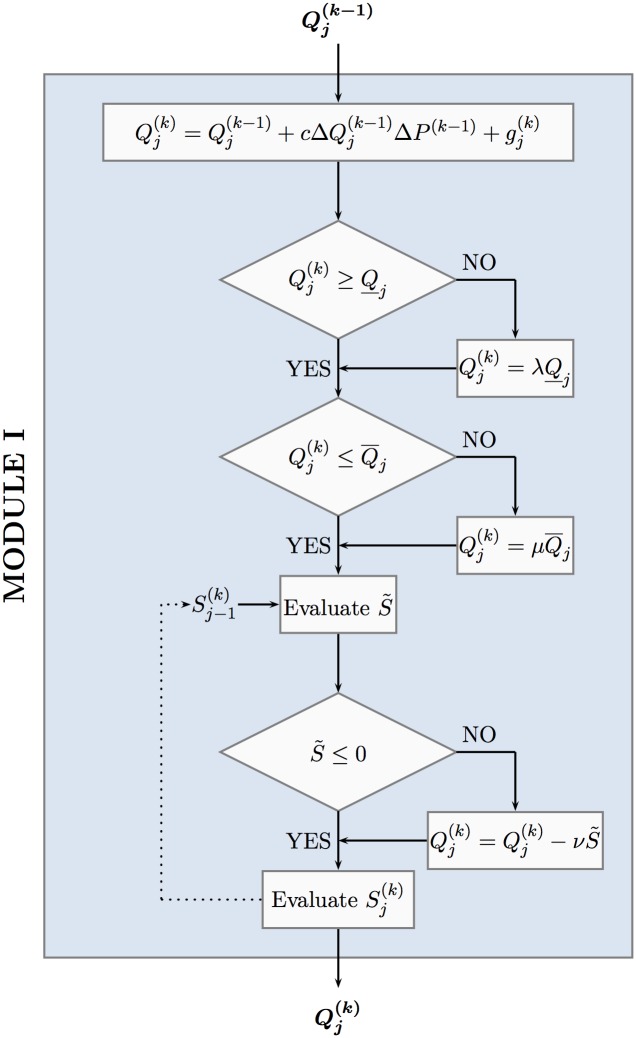
Flow chart of MODULE I.

The ALOPEX stochastic iteration step, described in Eq (24), together with the above three *local* constraint rectifications, described in Eqs (28)–(32), constitute MODULE I of the proposed technique. Its data flow is depicted in Fig 5, while the corresponding algorithmic implementation is demonstrated in Fig 6 below through the procedural flow chart that follows. One may easily observe the pipelined mode of execution through MODULE I.

∘ *Rectification for the toe constraint violation*

The strategy of rectifying the pumping rates of the wells in case of toe constraint violation unfolds in two stages. More specifically, let us assume that all values $Q_{j}^{\left(k\right)}$, *i* = 1, …, *M* have been evaluated by means of the ALOPEX main step Eq (24) and have been further rectified by relations Eqs (28), (29) and (32) whenever a corresponding violation has occurred. Then, for each index *p* ∈ {1, 2, …, *M*} such that a toe constraint violation has occurred pertaining to the corresponding *p*-well, namely

$$x_{\tau ,p}>x_{p}-\left(r_{c}+d_{s}\right)\;\;,$$ (33)

the first stage of rectification is implemented by

$$Q_{p}^{\left(k\right)}=\zeta_{p}Q_{p}^{\left(k\right)}\;\;,$$ (34)

where 0 < *ζ*_*p*_ < 1 is a real parameter.

**Fig 7:**
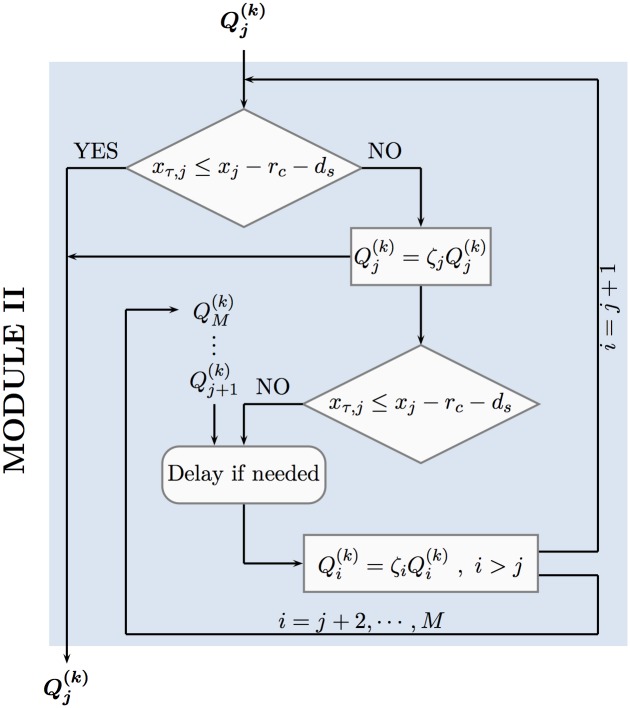
Chart and Data Flow through MODULE II.

In case the above correction does not resolve the toe violation problem occurred, and relation Eq (33) still holds, then the second stage of rectification is activated and implemented by

$$Q_{i}^{\left(k\right)}=\zeta_{i}Q_{i}^{\left(k\right)}\;\;\text{for}\;\text{each}\;\;i\in \left\{1,2,\ldots ,M\right\}\;\;\text{such}\;\text{that}\;\;\;\;x_{i}>x_{p}\;\;,$$ (35)

where 0 < *ζ*_*i*_ < 1 are real parameters. The two stages of the tow constraint rectification strategy, described above, is implemented algorithmically through MODULE II. Its procedural flow chart and data flow are depicted in Fig 7 that follows.

∘ *Rectification for the potential constraint violation*

A similar simple strategy may also be applied for rectifying the pumping rates of the wells in case of the potential constraint violation. More specifically, let us assume that all values $Q_{j}^{\left(k\right)}$, *i* = 1, …, *M* have been evaluated by means of the ALOPEX main step Eq (24) and have been further rectified by relations Eqs (28), (29), (32) and (34)–(35) whenever a corresponding violation has occurred. Then, for each index *p* ∈ {1, 2, …, *M*} such that a potential constraint violation has occurred pertaining to the corresponding *p*-well, namely

$$\phi ({\tilde{x}}_{p},y_{p})\leq 0\;\;,$$ (36)

where ${\tilde{x}}_{p}\approx x_{p}$ to avoid the singularity points of *ϕ*(*x*, *y*) at the well coordinates, then the value of $Q_{p}^{\left(k\right)}$ is being rectified by

$$Q_{p}^{\left(k\right)}=\xi Q_{p}^{\left(k\right)}\;\;\text{for}\;\;p\in \left\{1,2,\ldots ,M\right\}\;\;,$$ (37)

where *ξ* < 1 is a real parameter.

At this point we would like to conclude this section by means of the following remarks:

**Remark 1—Stopping Criterion** Besides our primary purpose to demonstrate the asymptotic converge properties of the stochastic constrained optimization strategy, described in the previous paragraphs, we also introduce an effective stopping criterion of the whole iterative process. The stochastic nature of the optimization procedure led us to use a combination of the standard deviation *σ*_Λ_ of the objective function’s values in the last Λ iterations (with Λ relatively small) and the differential of the objective function’s mean value *μ*_Λ_ in a window of the last 2Λ iterations. To be more specific, let us define the windowed mean value *μ*_Λ_ and standard deviation *σ*_Λ_ as

$$\mu_{\Lambda }=\frac{1}{\Lambda }\overset{k}{\underset{i=k-\Lambda }{\sum }}P^{\left(i\right)}\text{ and }\sigma_{\Lambda }=\sqrt{\frac{1}{\Lambda }\overset{k}{\underset{i=k-\Lambda }{\sum }}{\left(P^{\left(i\right)}-\mu_{\Lambda }\right)}^{2}}\;,$$ (38)

where *k* denotes the current iteration and *k* − 2Λ > 0. Then, for positive small tolerances *ϵ* and *δ*, whenever the value of the boolean expression

$$\left(\sigma_{\Lambda }<\epsilon \right)\wedge \left(\Delta \mu_{\Lambda }<\delta \right)$$ (39)

is true, where Δ*μ*_Λ_ = ∣*μ*_Λ_ − *μ*_2Λ_∣ with $\mu_{2\Lambda }=\frac{1}{\Lambda }\sum_{i=k-2\Lambda }^{k-\Lambda }P^{\left(i\right)}$, all values of the objective function are in a narrow neighborhood of their mean value while the rate of change of the mean values of the objective function tends to zero. Thus, assuming also that all constrains are satisfied, the optimization process may be effectively terminated. Although outside of the scope of this work, we point out that the above strategy may also be coupled with slope related criteria.

**Remark 2—Feedback amplitude** The value of the parameter *c*_*k*_ which, together with the term Δ*P*^(*k*−1)^ in Eq (24), determines the amplitude of the ALOPEX feedback mechanism, owes to honor simplicity and low cost computations, main characteristics of the ALOPEX process. For this, recall relation Eq (24) and observe that, in the absence of noise, one obtains

$$Q^{\left(k\right)}=Q^{\left(k-1\right)}+c_{k}\Delta P^{\left(k-1\right)}\left(Q^{\left(k-1\right)}-Q^{\left(k-2\right)}\right)\;,$$ (40)

and by extension or a Taylor expansion of *S*(***Q***) in Eq (13), we also have

$$S\left(Q^{\left(k\right)}\right)=S\left(Q^{\left(k-1\right)}\right)+c_{k}\Delta P^{\left(k-1\right)}\left(S\left(Q^{\left(k-1\right)}\right)-S\left(Q^{\left(k-2\right)}\right)\right)\;.$$ (41)

Furthermore, observing that relation Eq (17) implies

$$\Delta P^{\left(k-1\right)}\left(S\left(Q^{\left(k-1\right)}\right)-S\left(Q^{\left(k-2\right)}\right)\right)>0\;,$$

it is evident that

$$S\left(Q^{\left(k\right)}\right)=S\left(Q^{\left(k-1\right)}\right)+c_{k}|\Delta P^{\left(k-1\right)}||S\left(Q^{\left(k-1\right)}\right)-S\left(Q^{\left(k-2\right)}\right)|$$ (42)

and

$$c_{k}\geq 0\;⟹\;S\left(Q^{\left(k\right)}\right)\geq S\left(Q^{\left(k-1\right)}\right)$$ (43)

within noise. Taking, now, into consideration the fact that the objective function *P*(***Q***) is normalized, the choice of

$$c_{k}=\frac{1}{\left|\Delta P^{\left(k-1\right)}\right|}\geq 1$$ (44)

allows the feedback term to effectively couple the noise term and guide the random walk and, therefore, accelerates the process considerably.

**Remark 3—Noise amplitude** Certainly, several different strategies may be adopted for the effective determination of an appropriate noise interval. Traditionally, ALOPEX algorithms are combined with constant amplitude noise intervals, such as, for example,

$$g_{j}^{\left(k\right)}=\gamma {\bar{Q}}_{j}X_{j}^{\left(k\right)}\;,$$

where $g_{j}^{\left(k\right)}$ denotes the noise term in Eq (24), 0 < *γ* < 1 represents a small percentage of ${\bar{Q}}_{j}$, and $X_{j}^{\left(k\right)}$ denotes a random variable with values uniformly distributed in an interval G, such as G=(-0.5,1). The above rule has been successfully used in our simulations. In this work, we slightly deviate from the above rule and combine ALOPEX with varying amplitude noise interval. More precisely, we considered the noise interval

$$g_{j}^{\left(k\right)}=\gamma {\bar{Q}}_{j}^{\left(k-1\right)}X_{j}^{\left(k\right)}\;,$$ (45)

which takes into consideration the current values of the pumping parameters and implements an “annealing” strategy for the compromised front (that is, closest to the coast) wells. This varying amplitude noise interval proved to be at least as effective as the constant amplitude one and adopted in our simulations.

## Numerical simulations

Among the large number of simulations we performed to test the stochastic pumping management process, described in the previous sections, in this section we include the results of selected numerical simulations involving mainly low number of pumping wells, that better illustrate the qualitative characteristics of the sea front as well as the behavior of pumping management process.

The test case aquifer, used in all experiments, approximates a known real case aquifer at Vathi area of Kalymnos island in Greece (cf. [14, 15]) and is considered to be a rectangular aquifer with one barrier at the sea and three impermeable barriers on the other sides (see Fig 3). Its characteristics are reported in the Table 1 that follows.

**Table 1:** Aquifer and well characteristics.

*Aquifer characteristics*	*Well characteristics*
*L* = 7000*m*	*r*_*c*_ = 300*m*
*W* = 3000*m*	*d*_*s*_ = 100*m*
*K* = 100*m*/*day*	$\;{\bar{Q}}_{A}=10000m^{3}/day$
*q* = 1.23*m*^2^/*day*	$\;{\bar{Q}}_{j}=2500m^{3}/day,\;\;j=1,\ldots ,M$
*d* = 25*m*	$\;{\underline{Q}}_{j}=200m^{3}/day,\;\;j=1,\ldots ,M$
*N* = 30*mm*/*year*	*M* : *number of aquifer wells.*

The values of the pumping management parameters are included in Table 2 and have been kept constant in all numerical simulations. For their determination we have implemented a uniform strategy of ±5% correction. Namely, the value of any control variable, obtained after the enforcement of the penalty described in relations Eqs (28) or (32), is increased by 5%, while, after the enforcement of the penalties described in relations Eqs (29) or (34)–(35) or (37), is decreased by 5%. Therefore, the values of the corresponding parameters in the penalty relations Eqs (28)–(37) are 1±0.05 as reported in Table 2 that follows. The value of 5% has been chosen to maintain balance between two different targets. Namely, on one hand it has to be large enough to resolve the associated constraint violations and make the penalty system effective and, on the other hand, it has to be adequately small to allow the ALOPEX stochastic algorithm to guide the optimization process and converge within small fluctuations. Furthermore, as it pertains to the stopping criterion parameters in Eq (39), we remark that the optimization process terminates whenever all values of the objective function, during the last Λ iterations fluctuate around their mean value by a distance of at most *ϵ* = 10^−2^, an indication that the system converges at least locally to a value, and at the same time two consecutive windowed mean values of the objective function have at least three identical decimal points (*δ* = 10^−4^).

**Table 2:** Pumping management parameters.

*Parameter*	*Value*
λ in Eq (28) and *ν* in Eq (32)	1.05
*μ* in Eq (29), *ζ* in Eqs (34)–(35) and *ξ* in Eq (37)	0.95
Λ, *ϵ* and *δ* in Eq (39)	20, 10^−2^ and 10^−4^

All experiments were conducted on an Intel quad-core i7 2.6Mhz PC with 8 Gb DDR3 RAM using MATLAB environment.

### Simulation Cases and Optimization Profiles

The results from two groups of simulation cases are reported in this section. The first group includes four artificial test cases characterized by two or three pumping wells, located at positions (in meters) (*x*_*i*_, *y*_*i*_) (see Table 3), which, besides from illustrating the qualitative characteristics of the sea front, are used as well to test the optimization algorithm’s ability to sense small differences of the data profile and reflect them in the proposed pumping plan. The second group includes two test cases from the literature, characterized by five and eleven (cf. [14, 15] respectively) pumping wells (see Table 4), used for comparison purposes.

**Table 3:** Well Coordinates for the first group of simulation cases.

*Well Coordinates*	*Simulation Cases*			
	*Ia*	*Ib*	*IIa*	*IIb*
(*x*_1_, *y*_1_)	(2350, 800)	(1400, 800)	(2200, 500)	(2200, 1100)
(*x*_2_, *y*_2_)	(2400, 2150)	(2350, 2150)	(2200, 1600)	(3500, 2450)
(*x*_3_, *y*_3_)	-	-	(3500, 2450)	(5000, 500)

**Table 4:** Well Coordinates for the second group of simulation cases.

*Test Cases*	*Well Coordinates*					
	(*x*_1_, *y*_1_)	(*x*_2_, *y*_2_)	(*x*_3_, *y*_3_)	(*x*_4_, *y*_4_)	(*x*_5_, *y*_5_)	(*x*_6_, *y*_6_)
*IIIa*	(2657, 1572)	(3353, 2200)	(3932, 975)	(4632, 2470)	(4873, 1586)	-
*IIIb*	(2035, 1120)	(2390, 785)	(2445, 2160)	(2850, 1905)	(2875, 1600)	(3665, 1540)
	(*x*_7_, *y*_7_)	(*x*_8_, *y*_8_)	(*x*_9_, *y*_9_)	(*x*_10_, *y*_10_)	(*x*_11_, *y*_11_)	-
*IIIb*	(3895, 1400)	(4200, 1460)	(4505, 1795)	(4530, 1460)	(5090, 1710)	-

In our simulations we consider two different optimization profiles depending on whether or not we make use of the potential constraints. Namely,

- *Profile 1:* The ALOPEX is coupled by the constraints described in Eq (23)
- *Profile 2:* The ALOPEX is coupled by the constraints described in Eqs (22) and (23).

*Profile 1* is the constraint profile adapted in all simulations used to demonstrate the performance of the ALOPEX optimization algorithm. *Profile 1* is being reinforced, in the sequel, by the potential constraints, hence evolves to *Profile 2*, in order to report on its effect in resolving stability issues. To avoid the singularities of *ϕ*(*x*, *y*) at the well coordinates (*x*_*i*_, *y*_*i*_) in implementing the potential constraint Eq (22), the *x*-coordinate *x*_*i*_ is being approximated by $x_{i}\approx {\tilde{x}}_{i}=x_{i}-1$, as we have already mentioned in Eq (36).

### ALOPEX Pumping Management Performance

**Fig 8:**
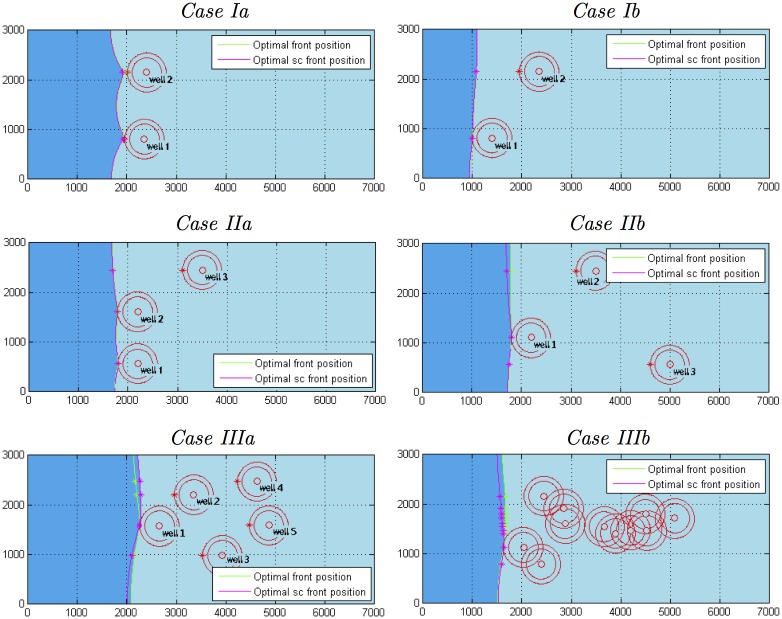
Optimal positions of the toe interface contour line for all simulation case.

**Fig 9:**
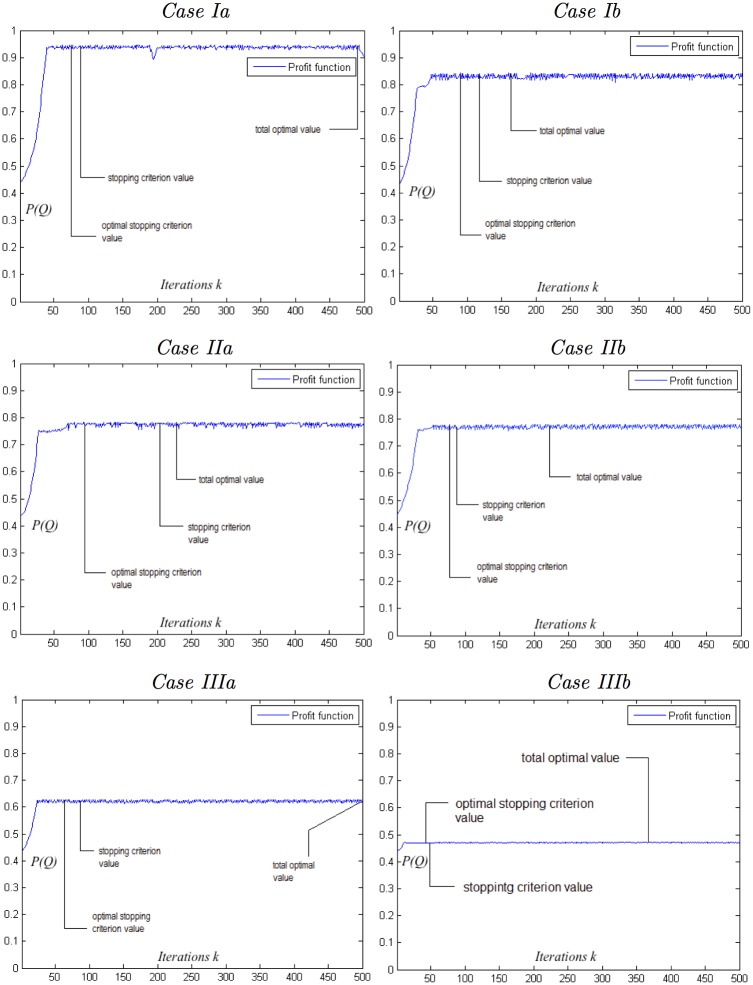
Evolution of the objective/profit function *P*(*Q*^(*k*)^) for all simulation cases.

**Fig 10:**
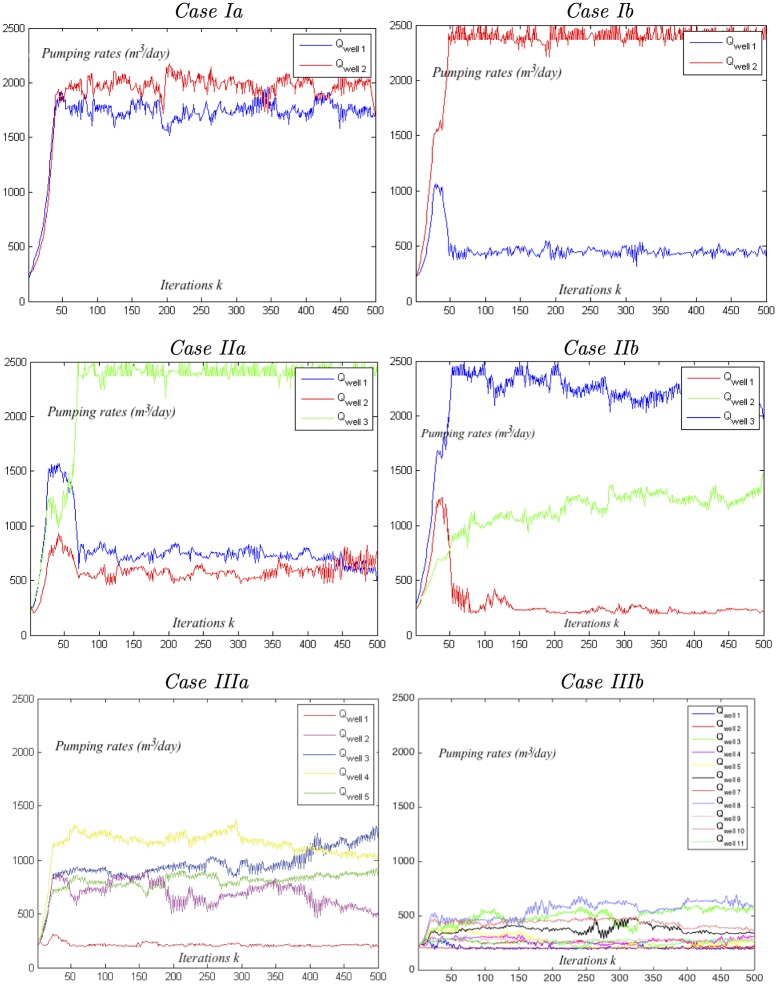
Evolution of the well pumping rates $Q_{j}^{\left(k\right)}\;\left(m^{3}/day\right)$ for all simulation cases.

The main results pertaining to the performance of the ALOPEX pumping management process for all simulations cases, when constraint *Profile 1* is implemented, are summarized in Tables 5–7 and Figs 8–10.

**Table 5:** ALOPEX pumping management performance: Optimal Parameter Values.

*Control Parameters*	*Optimal Values (within 500 iterations)*					
	*Ia*	*Ib*	*IIa*	*IIb*	*IIIa*	*IIIb*
*k* *(# iter.)*	492	160	228	221	500	364
*P*(***Q***^(*k*)^)	0.94605	0.84257	0.78285	0.78065	0.62769	0.47213
$Q_{1}^{\left(k\right)}$	1783.52	431.63	752.67	206.63	202.27	200.11
$Q_{2}^{\left(k\right)}\;|\;Q_{7}^{\left(k\right)}$	2038.97	2498.93	548.26	1254.18	504.38	207.41 | 205.78
$Q_{3}^{\left(k\right)}\;|\;Q_{8}^{\left(k\right)}$	-	-	2488.18	2306.97	1303.05	597.52 | 623.30
$Q_{4}^{\left(k\right)}\;|\;Q_{9}^{\left(k\right)}$	-	-	-	-	1047.07	365.97 | 207.62
$Q_{5}^{\left(k\right)}\;|\;Q_{10}^{\left(k\right)}$	-	-	-	-	912.85	345.26 | 413.77
$Q_{6}^{\left(k\right)}\;|\;Q_{11}^{\left(k\right)}$	-	-	-	-	-	286.40 | 223.07
*S*(***Q***^(*k*)^)	3822.49	2930.56	3789.11	3767.78	3969.62	3676.21
Time (sec)	1.97	1.31	2.28	3.48	7.87	17.36

**Table 6:** Implementing the Stopping Criterion: ALOPEX Optimal Parameter Values.

*Control Parameters*	*Optimal Values (within k_SC_ iterations)*					
	*Ia*	*Ib*	*IIa*	*IIb*	*IIIa*	*IIIb*
*k*|*k*_*SC*_	75|84	90|116	94|204	77|85	63|84	47|50
*P*(***Q***^(*k*)^)	0.94565	0.84233	0.78252	0.77793	0.62753	0.47094
$Q_{1}^{\left(k\right)}$	1793.04	434.80	769.21	293.57	206.41	314.07
$Q_{2}^{\left(k\right)}\;|\;Q_{7}^{\left(k\right)}$	2024.97	2494.08	530.57	1004.77	720.10	302.80 | 461.57
$Q_{3}^{\left(k\right)}\;|\;Q_{8}^{\left(k\right)}$	-	-	2486.10	2443.32	923.57	400.27 | 278.70
$Q_{4}^{\left(k\right)}\;|\;Q_{9}^{\left(k\right)}$	-	-	-	-	1278.27	439.21 | 330.94
$Q_{5}^{\left(k\right)}\;|\;Q_{10}^{\left(k\right)}$	-	-	-	-	838.96	283.75 | 315.99
$Q_{6}^{\left(k\right)}\;|\;Q_{11}^{\left(k\right)}$	-	-	-	-	-	224.32 | 284.63
*S*(***Q***^(*k*)^)	3818.01	2928.88	3785.88	3741.67	3967.30	3636.25
Time (sec)	0.33	0.30	0.93	0.59	1.32	1.74

**Table 7:** Coordinates *x*_*τ*_ (*meters*) of the toe critical points for all simulation cases.

*Toe Coordinates*	*Simulation Cases*					
	*Ia*	*Ib*	*IIa*	*IIb*	*IIIa*	*IIIb*
*x*_*τ*,1_	1925.40	999.93	1793.46	1799.92	2257.70	1633.72
*x*_*τ*,2_ | *x*_*τ*,7_	1957.20	1076.37	1793.33	1784.22	2207.14	1580.56|1654.83
*x*_*τ*,3_ | *x*_*τ*,8_	-	-	1699.48	1746.52	2155.85	1664.62|1657.12
*x*_*τ*,4_ | *x*_*τ*,9_	-	-	-	-	2177.09	1670.06|1668.15
*x*_*τ*,5_ | *x*_*τ*,10_	-	-	-	-	2256.57	1662.05|1657.12
*x*_*τ*,6_ | *x*_*τ*,11_	-	-	-	-	-	1659.96|1665.72
${\bar{x}}_{1}-x_{\tau ,1}$	24.60	0.07	6.54	0.08	0.49	1.28

To be more precise, aiming in testing ALOPEX asymptotic behavior, Table 5 includes the optimal values of the main parameters involved in the pumping management process obtained after 500 iterations, that is the values of the parameters that maximize the objective function

$$\max_{1\leq k\leq 500}P\left(Q^{\left(k\right)}\right)$$

and do not violate any of the constraints described in Eq (23).

To test the effectiveness of the stopping criterion, introduced in the previous section, Table 6 includes the optimal values of the main parameters obtained within *k*_*SC*_ iterations, that is the values of the parameters that maximize the objective function

$$\max_{1\leq k\leq k_{SC}}P\left(Q^{\left(k\right)}\right)$$

and do not violate any of the constraints described in Eq (23), where *k*_*SC*_ denotes the smallest iteration number for which the stopping criterion relation Eq (39) holds.

Observe that the optimal values of *P*(***Q***^(*k*)^) and *S*(***Q***^(*k*)^) included in Table 6 are practically the same to the corresponding values included in Table 5. Furthermore, the computational time included in Table 6 corresponds only to a small fraction of the computational time included in Table 5. These properties indicate the effectiveness of the stopping criterion used to terminate the ALOPEX optimization process.

The fact that the differences, between the asymptotic and stopping criterion optimal values, are small also yields small differences in the associated contours of the sharp interface between saltwater and freshwater at the bottom of the aquifer as depicted in Fig 8.

Table 7, that comes along with Fig 8, contains the *x*-coordinates *x*_*τ*,*i*_ of the toe critical points (*x*_*τ*,*i*_,*y*_*i*_) that correspond to the asymptotic optimal values (included in Table 5) of all simulation cases. The coordinates of the critical points that correspond to the stopping criterion optimal values are very close to the ones reported in Table 7, as may easily be verified by inspection of Fig 8, and therefore have been omitted.

An observation that merits highlighting is that the optimal position of the saltwater-freshwater interface, produced by the ALOPEX pumping management process and reported in Fig 8, remains very close to the perimeter of the guarding circular area of the frontal (closest to the sea) compromised well (*w*_1_) for all simulation cases. The exact difference of ${\bar{x}}_{1}-x_{\tau ,1}$, where ${\bar{x}}_{1}=x_{1}-\left(r_{c}+d_{s}\right)$, is reported in the last row of Table 7 and verifies the observation.

Figs 9 and 10, that follow, further explore the behavior of the ALOPEX stochastic optimization process. Fig 9 depicts the evolution of the objective function *P*(***Q***^(*k*)^), with respect of *k*, for all simulation cases. Observe that, in all cases, ALOPEX drives expeditiously the objective function *P*(***Q***) to its, restricted by the constrains, maximum value and remains close to it within relatively small amplitude fluctuations. Fig 10 depicts the evolution of the well pumping rates $Q_{i}^{\left(k\right)}\;\left(m^{3}/day\right)$, with respect of *k*, for all simulation cases. Observe that, the balance between algorithm’s deterministic terms (feedback and constrains) on one hand, and the stochastic term on the other, in order to achieve convergence within small fluctuations of the objective function around its maximum, is being achieved by several different motifs as it pertains to the behavior of the control variables *Q*_*i*_. For instance, the value of a control variable may converge within fluctuations at specific leveled value, as it happens for both variables of Case Ib in Fig 10. In cases, though, such as Cases Ia and IIa, in which local characteristics of the wells (*x*-coordinates and distance from the sea, minimum and maximum pumping rates) force their pumping rates to be close enough, it is possible to witness an “interweave” behavior during the optimization process. In Case Ia this happens only momentarily, as the algorithm is sensitive enough to realize even small differences (in the *x*-coordinates in this case) that affect the optimal value and “correct” its way to determine it.

Finally, as it pertains to the stability of the optimization results presented above, Table 8, that follows, contains the *x*-coordinates *x*_*s*,*i*_ (see Fig 2) of the stagnation points (*x*_*s*,*i*_, *y*_*i*_), *i* = 1, …, *M*, that correspond to the asymptotic optimal values (included in Table 5) of all simulation cases. As it can be verified by direct calculation, the difference *x*_*s*,1_ − *x*_*τ*,1_, reported in the last row of Table 8, satisfies

$$\Delta x_{s\tau }\equiv x_{s,1}-x_{\tau ,1}=\min_{1\leq i\leq M}\left(x_{s,i}-x_{\tau ,i}\right)>0\;,$$

hence all stagnation points remain between their associated critical toe interface points and the corresponding wells and, therefore, the optimization results satisfy the necessary stability requirement.

**Table 8:** Coordinates *x*_*s*_ (*meters*) of the stagnation points for all simulation cases.

*Toe Coordinates*	*Simulation Cases*					
	*Ia*	*Ib*	*IIa*	*IIb*	*IIIa*	*IIIb*
*x*_*s*,1_	2004.61	1325.10	2015.75	2118.78	2535.07	1964.57
*x*_*s*,2_|*x*_*s*,7_	2014.36	1989.32	2040.36	3123.34	3133.94	2336.66|3854.98
*x*_*s*,3_|*x*_*s*,8_	-	-	3055.61	4507.12	3557.72	2252.04|4110.03
*x*_*s*,4_|*x*_*s*,9_	-	-	-	-	4432.84	2774.37|4478.70
*x*_*s*,5_|*x*_*s*,10_	-	-	-	-	4737.71	2794.60|4486.14
*x*_*s*,6_|*x*_*s*,11_	-	-	-	-	-	3604.17|5065.18
*x*_*s*,1_ − *x*_*τ*,1_	79.21	325.16	222.29	318.86	277.37	330.86

Observe, however, that as the magnitude of Δ*x*_*sτ*_ is relatively small, the optimal results presented in Table 5, although stable, are sensitive to small augmentative perturbations. For instance, increasing the optimal values of all control variables *Q*_*i*_ reported for Cases IIIa and IIIb in Table 5 by 1.10% and 3.85%, respectively, would lead to unstable results. Sensitivity issues of the optimization results are further discussed in the section that follows.

### Validity/Stability Issues of the Optimization Results

As we have already mentioned, results have shown that the sharp-interface approximation is reasonable in regional scale problems when the transition zone is narrow relative to the scale of the problem (cf. [9, 15]). The validity study of the sharp-interface approximation, performed in [9], has concluded that the width of the transition zone decreases for:

- higher values of the recharge parameter *N*,
- lower values of the horizontal hydraulic conductivity parameter *K*,
- lower distances *x*_*w*_ of the wells from the coast,
- higher values of the aquifer’s height *d*.

In this section, in an attempt to create scenarios that enhance the validity of the optimization results presented in the previous section, we consider the model problems described by the Test Cases IIIa and IIIb and investigate validity, sensitivity and stability issues of the optimization results as it pertains to the recharge and hydraulic conductivity parameters *N* and *K*.

**Fig 11:**
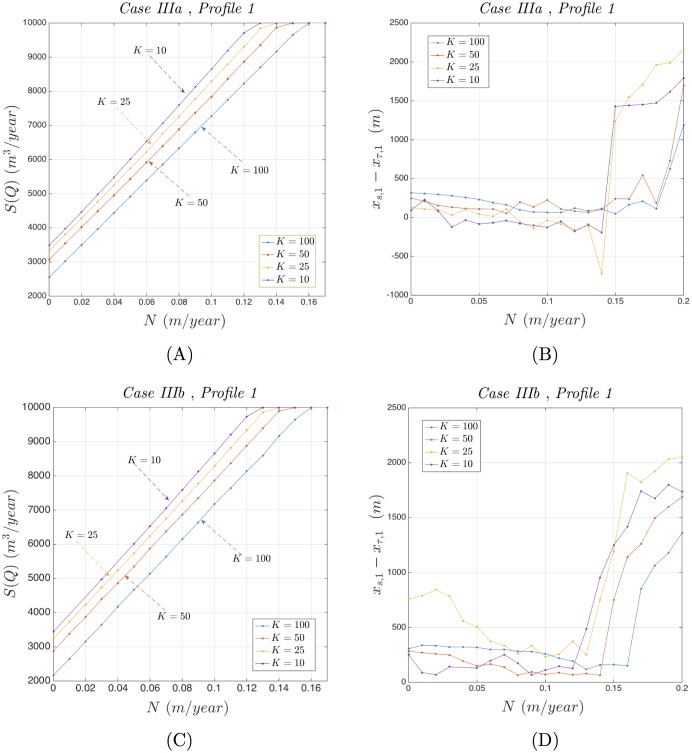
Behavior of *S*(*Q*) and Δ*x*_*sτ*_ under Profile 1. Dependence of *S*(***Q***) and Δ*x*_*sτ*_ on *N* and *K* for Test Cases IIIa and IIIb using Profile 1.

**Fig 12:**
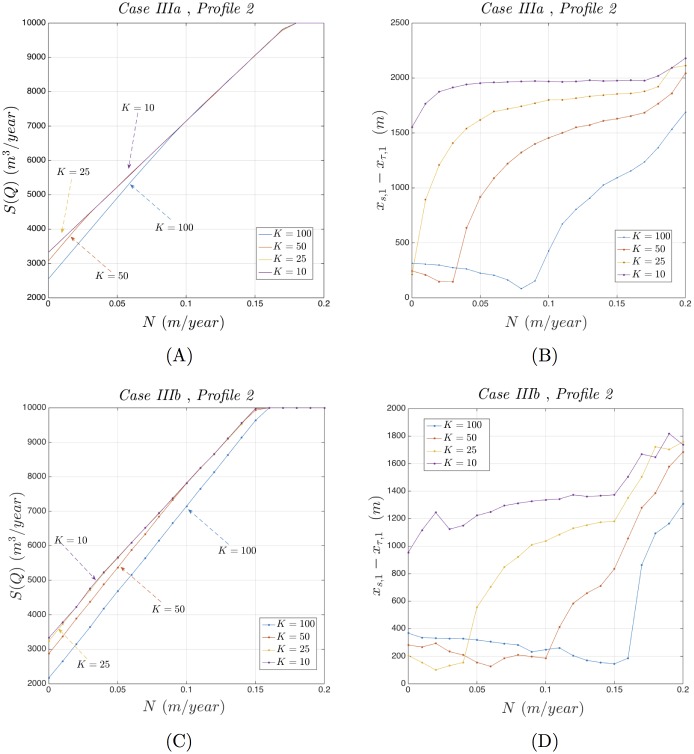
Behavior of *S*(*Q*) and Δ*x*_*sτ*_ under Profile 2. Dependence of *S*(***Q***) and Δ*x*_*sτ*_ on *N* and *K* for Test Cases IIIa and IIIb using Profile 2.

The optimization results obtained are summarized through Figs 11 and 12 and refer to the usage of optimization Profiles 1 and 2 respectively. Both figures explore the dependence of the optimal total pumping rate *S*(***Q***), and its associated stability related difference Δ*x*_*sτ*_ = *x*_*s*,1_ − *x*_*τ*,1_, on the parameters *N* and *K*, while keeping the rest of the parameters fixed at their values included in Tables 1 and 2.

Inspecting Fig 11, that refers to the usage of the optimization Profile 1, one may observe that:

- The optimal pumping rate *S*(***Q***), in both test cases IIIa and IIIb (Fig 11A and 11C respectively), increases linearly with respect of *N* and decreases with respect of *K*. After a relatively large value of *N*, *S*(***Q***) reaches the aquifer’s maximum pumping capability ${\bar{Q}}_{A}=10000m^{3}/day$ activating the corresponding constraint.
- The stability related difference Δ*x*_*sτ*_, as it pertains to the test case IIIb (Fig 11D), remains positive and therefore the necessary stability criterion is being satisfied for all values of *N* and *K*. Observe, however, that as *N* increases and *K* decreases its magnitude is getting relatively close to zero. Therefore, the corresponding optimization results are sensitive to small augmentative perturbations and close to instability regions. As it pertains to the test case IIIa (Fig 11B) observe that, for large intervals of *N* and for *K* = 10, 25, the difference Δ*x*_*sτ*_ becomes negative hence the corresponding optimization results are unstable.

In summary, one may remark that, using optimization Profile 1, higher values of *N* and lower values of *K* decrease the width of the transition zone and increase the total pumping rate but at the risk of instability.

Utilization of optimization Profile 2, namely augmenting the set of constraints described in Eq (23) -and used in Profile 1- by the potential constraint Eq (22), may help to overcome the above instability problems.

Indeed, inspecting Fig 12, that refers to the usage of the optimization Profile 2, one may observe that:

- The optimal pumping rate *S*(*Q*), in both test cases IIIa and IIIb (Fig 12A and 12C respectively), increases with respect of *N*, while reaches a limiting value as *K* decreases. After a relatively large value of *N*, *S*(*Q*) reaches the aquifer’s maximum pumping capability ${\bar{Q}}_{A}=10000m^{3}/day$ activating the corresponding constraint.
- The stability related difference Δ*x*_*sτ*_, as it pertains to both test cases IIIa and IIIb (Fig 12B and 12D respectively), remains positive and therefore the necessary stability criterion is being satisfied for all values of *N* and *K*. Moreover, in all practical cases there exists a non-negative number *N*_*K*_ ≥ 0 such that for *N* > *N*_*K*_ the value of Δ*x*_*sτ*_ increases, as *N* increases and *K* decreases, improving stability.

In summary, one may remark that, using optimization Profile 2, higher values of *N* and lower values of *K* decrease the width of the transition zone, increase the total pumping rate up to a limiting upper bound and improves stability. Therefore, utilization of optimization Profile 2 should be preferred over optimization Profile 1 as it may improve both validity and stability of the optimization results.

To conclude this section we point out that a complete sensitivity analysis is necessary, since the validity and stability of the optimization results depend on a number of other parameters too. This, however, exceeds the purpose of this work and will be presented elsewhere.

## Conclusions

This work presents a new version of the ALOPEX stochastic optimization algorithm and studies its performance when applied on analytical models of saltwater intrusion of coastal aquifers. The objective of the optimization problem was to maximize the volume of the fresh groundwater pumped from the aquifer subject to a set of constraints that prevent the intrusion of the saltwater.

The mathematical formulation of the saltwater intrusion problem was based on the work presented by Mantoglou [14] using an analytical model that is based on the sharp interface approach and the use of the Ghyben-Herzberg relation.

During the course of our study, we:

- Determined the amplitude of the algorithm’s feedback and noise terms that accelerate the process considerably,
- Presented a penalty system to implement the constraints,
- Introduced a stopping criterion for termination of the iterative stochastic process.

The model case aquifer simulates the coastal aquifer at Vathi on the island of Kalymnos in Greece. The model aquifer’s characteristics (Table 1) are identical to the ones used in [14]. Several test cases, consisting of two, three, five and eleven wells, were examined. Utilizing the proposed ALOPEX stochastic algorithm and for all test case problems we:

- Derived the optimal pumping rates (Table 5) of all active wells within a fixed large number of iterations (asymptotic case). The optimal results strongly compete the corresponding results from the literature while the CPU time for their computation varied from 1.97 sec to 17.36 sec.
- Tested the effectiveness of the stopping criteria. The corresponding optimal results obtained (Table 6) found in complete agreement with the corresponding asymptotic ones and computed at a fraction of the CPU time (from 0.33 sec to 1.74 sec).
- Explored the convergence profile of the objective function to verify that in all cases the algorithm drives expeditiously the objective function to its maximum and remains close to it within small amplitude fluctuations (Fig 9).
- Computed the coordinates of the critical points (Table 7) as well as the stagnation points (Table 8) to verify the stability of the optimal optimization results.

Finally, we investigated the dependence of stability of the optimization results on the recharge and hydraulic conductivity parameters *N* and *K*, and showed that the usage of the potential constraint profile (Profile 2) improves the stability of the optimization results (Fig 12).
